# When tasks cross the line: the mediating role of territoriality in the relationship between illegitimate tasks and self-interested voice

**DOI:** 10.3389/fpsyg.2026.1962135

**Published:** 2026-10-07

**Authors:** Qian Ruan, Cheng Liu

**Affiliations:** 1 Business School, Sichuan University, Chengdu, China; 2 Department of Management and Marketing, The Hong Kong Polytechnic University, Hong Kong, Hong Kong SAR, China

**Keywords:** conservation of resources theory, illegitimate tasks, perceived organizational politics, self-interested voice, territoriality, time urgency

## Abstract

Illegitimate tasks are increasingly prevalent in modern organizations. Prior research has established their harmful consequences and largely explained employee reactions through the lens of task and role incongruence, emphasizing legitimacy judgments and threats to occupational identity. However, the psychological mechanisms through which illegitimate tasks activate employees’ motivation to protect their resources remain largely underexplored. Drawing on conservation of resources theory, we explored how territoriality mediates the association between illegitimate tasks and self-interested voice. Additionally, time urgency and perceived organizational politics were identified as moderators. Using a three-wave, time-lagged design with two-week intervals, we collected data from 350 full-time employees, retained 306 valid responses, and tested seven hypotheses. Predictor, mediator, and outcome variables were measured at separate time points. Results suggested that illegitimate tasks were positively linked to territoriality, which was subsequently positively associated with self-interested voice. When both moderators were examined simultaneously, time urgency strengthened the positive effect of illegitimate tasks on territoriality and further amplified the indirect effects. The moderating effect of perceived organizational politics was not significant when examined jointly. This study advances theoretical understanding in the illegitimate tasks and voice literature while providing practical guidance for organizations seeking to optimize task allocation and reduce defensive employee responses.

## 1 Introduction

As organizations face enduring economic and technological pressures, increasing workplace demands have made illegitimate tasks a growing feature of modern organizational life (Sharma and Pandey, 2026). Illegitimate tasks are defined as unnecessary or unreasonable task demands that violate occupational role norms (Semmer et al., 2015) and have attracted growing scholarly attention due to their harmful consequences at both the individual and organizational levels (e.g., Zhao et al., 2022). Prior studies have linked illegitimate tasks to plenty of adverse employee outcomes, including reduced self-esteem (Eatough et al., 2016), heightened negative affect (Sonnentag and Lischetzke, 2018), and increased anger (Zhou et al., 2018). Beyond individual well-being, these tasks have also been linked to diverse behavioral responses, including emotional labor strategies (Cheng et al., 2026), quiet quitting (Ma et al., 2026) and counterproductive work behaviors (Liu et al., 2025; Zhao et al., 2022). Although scholarly attention to the psychological and behavioral consequences of illegitimate tasks has grown considerably (Ding and Kuvaas, 2023; Tian et al., 2026), less is known about the psychological processes through which illegitimate tasks activate employee’s resource-protective psychological state and influence workplace behaviors.

Drawing on conservation of resources theory (COR theory; Hobfoll, 1989), this study attempts to explore the following research questions: what role does employee’s resource-protective psychological state play in linking illegitimate tasks with subsequent reactive behavior? Moreover, how are these relationships moderated by the individual trait and the situational factor? COR theory states that individuals are fundamentally motivated to protect and accumulate valued resources, and that perceived resource loss or threat activates resource conservation tendencies (Halbesleben et al., 2014). Drawing on the theory, we argue that illegitimate tasks constitute a dual resource threat, depleting employees’ energy resources while encroaching upon their condition resources. Illegitimate tasks consume employees’ energy resources such as available work time and sustained attention, which are difficult to replenish quickly (Hobfoll, 1989; Semmer et al., 2015). They also threaten valued condition resources including occupational identity and role clarity (Hobfoll, 1989), which are central sources from which employees derive meaning and status at work (Ashforth and Kreiner, 1999; Caza et al., 2018). This dual threat to energy and condition resources together creates the motivational conditions for territoriality to be activated. Territoriality provides an important psychological perspective for understanding how employees respond when their workplace resources are compromised. Prior research has shown that perceived resource threat activates territorial cognitions in organizational contexts (Avey et al., 2009; Gardner et al., 2018; Venkataramani et al., 2024), and this study extends that logic to the illegitimate task context.

Territoriality refers to a unique, prevention-focused form of psychological ownership characterized by an external referent and defensiveness (Avey et al., 2009). Psychological ownership captures the general feeling that a target is “mine,” whereas territoriality is distinguished by its focus on potential external interference and its defensive nature. Territoriality reflects employees’ perceived need to mark, defend, and assert control over their work domains and resources when they believe that others may influence or encroach on what they regard as their own (Avey et al., 2009; Chen et al., 2023). When territoriality is activated, employees may take corresponding actions to protect their domains and resources, which can not only trigger defensive and even antagonistic behaviors toward colleagues and the organization, undermining teamwork and intra-organizational resource sharing, but also prompt various strategic behaviors aimed at consolidating and expanding their resource boundaries, with far-reaching consequences for the organization (Brown, 2009; Brown et al., 2005; Gardner et al., 2018; Venkataramani et al., 2024). Nevertheless, how territoriality is activated by specific work stressors and what behavioral responses it subsequently triggers remain in need of further theoretical elaboration and empirical examination (Brown, 2009; Ding and Kuvaas, 2023).

Territoriality not only reflects employees’ perceived need to defend their work domains and resources, but also shapes the strategic behaviors they adopt when resources are threatened (Brown et al., 2005). According to COR theory, when individuals experience actual resource loss or face the threat of resource loss, they take action to replenish depleted resources or prevent further loss (Hobfoll, 2001; Hobfoll et al., 2018). We argue that heightened territoriality may be associated with a particular form of proactive behavior, namely self-interested voice (SIV), which refers to communicative behavior in which employees actively express opinions on issues that directly concern their personal interests (Duan et al., 2021). Unlike covert self-interested behaviors such as concealment and non-cooperation (Connelly et al., 2012; Deutchman and Sullivan, 2018), self-interested voice involves an active expressive element whereby employees openly advocate for arrangements favorable to their own interests, such as favorable task assignments, recognition, or autonomy, in order to influence organizational decisions, making it a distinctive phenomenon situated at the intersection of voice behavior and self-interested behavior (Duan et al., 2021; Lou et al., 2023). By speaking up, employees may attempt to influence organizational arrangements in ways that serve their own interests, thereby restoring or protecting their resource reserves. Therefore, this study focuses on territoriality as the psychological mechanism linking illegitimate tasks with self-interested voice and further examines the boundary conditions under which this process is amplified.

According to conservation of resources theory, both personal traits and situational factors play important roles in shaping individual attitudes and behaviors (Hobfoll, 1989, 2001). Illegitimate tasks also pose a dual resource threat by depleting employees’ energy resources while encroaching upon their condition resources (Hobfoll, 1989; Semmer et al., 2015). We therefore identify time urgency as an individual boundary condition that may shape how employees respond to the depletion of energy resources and perceived organizational politics as a contextual boundary condition that may shape how employees interpret threats to condition resources. Time urgency reflects individuals’ sensitivity to time scarcity and resource loss (Burnam et al., 1975; Landy et al., 1991). Employees high in time urgency may perceive illegitimate tasks as stronger threats to their limited time resources. Perceived organizational politics (POP) is defined as employees’ perception that organizational decisions and resource allocations are driven more by informal power, favoritism, and political maneuvering than by objective or merit-based criteria (Ferris et al., 1996; Ferris and Kacmar, 1992). Under politically charged conditions, employees may be more likely to interpret illegitimate tasks as resource encroachment (Hochwarter et al., 2000), thereby strengthening territorial responses.

Using three-wave, time-lagged data from 306 full-time employees, this study tests a moderated mediation model linking illegitimate tasks to self-interested voice through territoriality. This study makes three contributions. First, this study advances research on illegitimate tasks by identifying territoriality as the underlying psychological mechanism linking illegitimate tasks to employee behavior, thereby introducing an explanatory perspective centered on prevention-focused psychological ownership. Second, by identifying self-interested voice as an outcome variable through which illegitimate tasks influence employee behavior via territoriality, this study contributes to the literature on both illegitimate tasks and voice behavior. Finally, by jointly examining time urgency and perceived organizational politics, this study provides a more differentiated account of the boundary conditions surrounding the proposed resource-protective process. Practically, this study also offers meaningful implications for organizations seeking to optimize task allocation and reduce defensive employee responses.

## 2 Theory and hypotheses development

### 2.1 Conservation of resources theory

COR theory posits that individuals faced with the threat of resource loss or actual resource loss are motivated to conserve their remaining resources and take protective action in response (Hobfoll, 2001). In organizational contexts, the resources employees value include not only energy resources such as time, effort, and attention, but also condition resources such as role boundaries and one’s positional role at work (Hobfoll, 1989). When these resources are unreasonably consumed or threatened, employees’ concern for resource protection becomes heightened, prompting them to take action to avoid further loss while also proactively building resource reserves to cope with future threats (Hobfoll et al., 2018).

Drawing on this theoretical foundation, we argue that illegitimate tasks not only consume additional time but also constitute an affront to employees’ positional and role legitimacy. Under such circumstances, employees are likely to experience heightened territoriality, reflected in a stronger perceived need to protect their work domains and resources from external interference (Avey et al., 2009; Chen et al., 2023). Consequently, when employees place greater emphasis on personal domain and control, they may become more attentive to organizational arrangements that directly affect their own interests and are more inclined to speak up in an attempt to influence those arrangements. Following this logic, this study proposes that illegitimate tasks may be positively associated with employees’ territoriality, thereby prompting employees to voice their concerns on issues related to their personal interests. At the same time, employees’ sensitivity to time as a resource and the political climate of their workplace further shape the extent to which illegitimate tasks are interpreted as resource threats. These factors therefore moderate the relationship between illegitimate tasks and territoriality and thereby influence the overall indirect effect (Figure 1).

**Figure 1:**
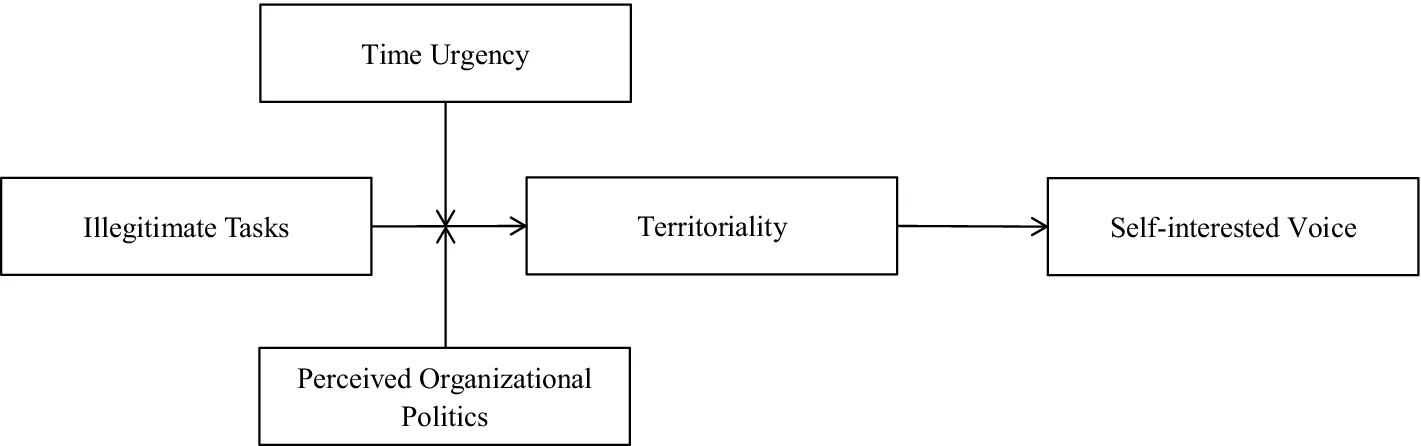
The proposed model.

### 2.2 Illegitimate tasks and territoriality

From a COR theory perspective, illegitimate tasks represent a dual resource threat. On the one hand, they require employees to expend additional time resources by imposing demands that employees perceive as wasteful and unwarranted (Semmer et al., 2015), while such energy resources are inherently limited for employees (Trougakos et al., 2015). On the other hand, they simultaneously threaten valuable condition resources (Hobfoll, 1989). Specifically, illegitimate tasks convey indirect signals of disrespect and degradation, threatening employees’ sense of professional identity and role clarity, all of which are important resources through which individuals derive meaning from their work (Eatough et al., 2016; Semmer et al., 2015). Consequently, the assignment of these tasks both depletes employees’ limited energy resources and threatens condition resources related to their sense of professional identity.

When faced with resource threats, individuals will activate psychological protective motivations to preserve existing resources and obtain additional resources to offset losses (Hobfoll, 1989; Hobfoll et al., 2018). Territoriality is a unique, prevention-focused form of psychological ownership characterized by an external referent and defensiveness (Avey et al., 2009). It becomes heightened when employees believe that others may influence or encroach on what they regard as their own. Employees therefore become concerned with protecting these targets and maintaining control over them (Chen et al., 2023). When illegitimate tasks erode employees’ sense of professional ownership and role legitimacy while simultaneously consuming additional time resources, employees may develop stronger psychological claims over their work domain, role boundaries, and time resources (Brown et al., 2005). Existing research consistently demonstrates that perceived resource threats activate territorial cognition in organizational contexts (Brown, 2009; Gardner et al., 2018; Venkataramani et al., 2024). Based on this reasoning, we propose the following hypothesis:

*H1:* Illegitimate tasks are positively related to employees’ territoriality.

### 2.3 The mediating role of territoriality

Self-interested voice refers to employees’ behavior of expressing opinions and communicating on issues that directly concern their personal interests (Duan et al., 2021). Unlike organization-oriented voice, self-interested voice can produce negative consequences, making it an unavoidable managerial concern (Duan et al., 2021). Although it is a form of self-interested behavior, self-interested voice differs from conventional self-interested behaviors. Traditional self-interested behaviors commonly involve concealing information, manipulating others, or refusing to cooperate (Connelly et al., 2012; Deutchman and Sullivan, 2018), whereas self-interested voice incorporates an active expressive element. Employees seek to change the status quo by voicing and offering suggestions. Although their motivation is to pursue personal gain and acquire resources, they nonetheless exhibit a tendency toward active engagement (Duan et al., 2021). This distinction positions self-interested voice as a phenomenon that sits between self-serving behavior and voice behavior, combining the expressive and open nature of voice with the self-centered orientation of self-oriented behavior (Duan et al., 2021; Lou et al., 2023). This combination makes self-interested voice a particularly compelling subject for examination within a COR theory framework.

Employees with higher territoriality are more sensitive to threats directed at their resource pool (Brown and Robinson, 2011; Venkataramani et al., 2024). As a defensively oriented form of psychological ownership, territoriality functions primarily to preemptively protect employees’ work domains and resource boundaries, manifesting as marking territory, adopting anticipatory defenses to deter encroachment, and employing reactionary defenses to prevent further infringement (Avey et al., 2009). Reactionary defenses are responsive behaviors implemented by organizational members after an attempted infringement (Brown and Robinson, 2011). We argue that self-interested voice is a form of reactionary defense driven by territoriality. Employees with higher territoriality may advocate for arrangements, policies, or decisions that favor their interests, such as favorable task assignments, recognition, or autonomy, with the aim of securing compensation in future resource allocations and preventing similar encroachments from recurring. Therefore, we posit that:

*H2a:* Territoriality is positively associated with self-interested voice.

Drawing on H1 and H2a, and grounded in COR theory, we propose that territoriality serves as a key mediating mechanism between illegitimate tasks and self-interested voice. Specifically, we argue that when employees perceive themselves as being assigned these tasks, they are more inclined to experience heightened territoriality, because such tasks consume additional time resources while simultaneously threatening their professional role clarity. In turn, this heightened territoriality motivates employees to engage in self-interested voice as a means of acquiring additional resources or preventing further resource loss. Therefore, we posit that:

*H2b:* Territoriality mediates the relationship between illegitimate tasks and self-interested voice, such that illegitimate tasks have a positive indirect effect on self-interested voice through territoriality.

### 2.4 The moderating role of time urgency

COR theory indicates that personal characteristics help shape individual attitudes and behaviors (Hobfoll, 1989). We argue that time urgency, as a personal characteristic, amplifies the effect of illegitimate tasks on territoriality. Specifically, time urgency reflects a stable personality tendency to treat time as a scarce resource and to be highly sensitive to its loss, manifesting as a persistent perception of time pressure and time-related anxiety (Burnam et al., 1975; Landy et al., 1991). Individuals high in time urgency tend to regard time as an exceptionally precious and non-renewable energy resource, maintaining heightened vigilance toward any demands that may consume their time (Conte et al., 1995). When illegitimate tasks impose additional time consumption, such employees are more likely to interpret this as a serious encroachment on their core resources, thereby triggering a stronger resource protection motivation (Halbesleben et al., 2014; Hobfoll, 1989). By contrast, employees low in time urgency are less sensitive to the time-related demands of these tasks, and the positive relationship between illegitimate tasks and territoriality is expected to be attenuated accordingly. Therefore, we posit that:

*H3a:* Time urgency moderates the effect of illegitimate tasks on territoriality, such that the effect is stronger when time urgency is higher.

Building on this, we argue that time urgency also conditions the indirect relationship between illegitimate tasks and self-interested voice via territoriality. When employees are higher in time urgency, the positive association between illegitimate tasks and territoriality is amplified, and consequently, the indirect pathway from illegitimate tasks to self-interested voice through territoriality is correspondingly strengthened. Therefore, we propose that:

*H3b:* Time urgency moderates the indirect effect of illegitimate tasks on self-interested voice through territoriality, such that the indirect effect is stronger when employees’ time urgency is higher.

### 2.5 The moderating role of perceived organizational politics

As noted earlier, illegitimate tasks are linked to both the depletion of employees’ energy resources and the perceived threat to their condition resources, including their sense of professional identity and role legitimacy (Hobfoll, 1989; Semmer et al., 2015). Meanwhile, the perceived intensity of condition resource threats is also moderated by situational factors. Perceived organizational politics, as an important perception of organizational context, shapes both the way employees interpret condition resource threats and the intensity of those interpretations (Hochwarter et al., 2000). Perceived organizational politics is conceptualized as employees’ subjective appraisal of the extent to which self-serving behaviors are present within the organization, reflecting the tendency of individuals or groups to pursue their own interests through strategic means (Ferris and Kacmar, 1992; Ferris et al., 1996). Such perceptions also reflect the view that organizational decisions and resource allocations are driven more by informal power, favoritism, and political maneuvering than by objective or merit-based criteria (Kacmar and Carlson, 1997). Against this backdrop, we argue that perceived organizational politics strengthens the positive impact of illegitimate tasks on territoriality.

First, a high level of POP signals the presence of self-serving power dynamics within the organization (Castanheira et al., 2022), which itself constitutes a situational stress that threatens condition resources. In such an environment, employees perceive that condition resources such as job security, career advancement opportunities, and role legitimacy are constantly at risk of being encroached upon, and that formal rules and procedures offer little protection, thereby creating a resource-threatening environment characterized by pervasive uncertainty (Chang et al., 2009; Ferris et al., 2002). Second, perceived organizational politics may provide an interpretive frame through which employees make sense of illegitimate task assignments. Perceived organizational politics reflects employees’ subjective assessment that self-serving behavior and informal influence characterize their work environment, rather than an objective record of political behavior (Ferris and Kacmar, 1992; Kacmar and Carlson, 1997; Ferris et al., 2002). Prior research further suggests that employees’ responses to illegitimate tasks depend partly on how they attribute the reasons for receiving such assignments (Wang and Jiang, 2023). In highly political environments, illegitimate tasks may be viewed as reflecting self-serving motives or attempts by powerful actors to shift unreasonable burdens onto others (Hochwarter et al., 2020; Wang and Jiang, 2023). Such an interpretation may make illegitimate tasks appear more intentional and more threatening to employees’ time resources and professional identities (Ding and Kuvaas, 2023; Semmer et al., 2015).

Therefore, we propose that when employees who perceive high levels of POP encounter illegitimate tasks, they may not only face the situational stress of condition resource threats inherent in a politically charged environment, but may also interpret illegitimate tasks through a more politically charged lens, an interpretation that may be associated with a stronger sense of territoriality (Brown et al., 2005; Hobfoll, 1989). In contrast, employees operating under conditions of low perceived organizational politics experience a comparatively limited degree of resource threat, and their territorial responses are correspondingly weaker. Thus, we propose that:

*H4a:* Perceived organizational politics moderates the effect of illegitimate tasks on territoriality, such that the effect is stronger when perceived organizational politics is higher.

Furthermore, we propose that perceived organizational politics also moderates the indirect effect of illegitimate tasks on self-interested voice through territoriality. When perceived organizational politics is high, the effect of illegitimate tasks on territoriality is strengthened, and in turn, the indirect effect of illegitimate tasks on self-interested voice via territoriality is also enhanced. Based on this reasoning, we propose that:

*H4b:* Perceived organizational politics moderates the indirect effect of illegitimate tasks on self-interested voice through territoriality, such that the indirect effect is stronger when employees’ perceived organizational politics is higher.

## 3 Methods

### 3.1 Sample and procedures

Full-time employees were recruited via Jishuyun, a reputable professional data collection platform in China that has been utilized in prior academic research (Kamil et al., 2025; Liu et al., 2026). Survey responses were collected anonymously, and each participant was assigned a unique identification code. The platform used single-use survey links and IP tracking mechanisms, which together prevented duplicate submissions and safeguarded the authenticity of each response.

A three-wave time-lagged design with two-week intervals was employed, separating the measurement of predictors, mediators, and outcomes across time to reduce common method variance concerns, while aligning the timing of variable assessment with the theorized sequence to support the proposed directional relationships (Kim et al., 2024). All variables were assessed at the individual level through employee self-reports. At Time 1, demographic information was collected alongside measures of illegitimate tasks, perceived organizational politics, and time urgency. At Time 2, employees’ territoriality was measured. At Time 3, we collected data on self-interested voice. Each wave incorporated attention check items; responses were linked across waves via unique identification codes, and only those participants who completed all three waves and passed every attention check were included in the final analysis.

In Wave 1, we obtained 350 valid questionnaires. After applying the same exclusion criteria across waves, namely failed attention checks and incomplete responses, 320 valid questionnaires were retained in Wave 2 and 306 in Wave 3. The final sample comprised 306 participants, yielding a valid response rate of 87.43%. Regarding demographics, 35.62% were female and the average age was 33.31 years. Moreover, 82.68% reported a bachelor’s degree or above. Position levels were as follows: 38.56% general staff, 40.20% frontline managers, 19.93% middle managers, and 1.31% senior managers.

### 3.2 Measures

All measures were administered in Chinese using Chinese-language versions that had been previously used and validated in Chinese samples. Relevant references for the Chinese versions are provided below for each measure. Participants rated all constructs on a 5-point scale (1 = *strongly disagree*, 5 = *strongly agree*).

#### 3.2.1 Illegitimate tasks

Following Liu et al. (2025), we used the 8-item scale of Semmer et al. (2010) to measure illegitimate tasks, comprising four items measuring unnecessary tasks and four items measuring unreasonable tasks. A sample item was “I need to do some tasks which should be done by someone else” (α = 0.94).

#### 3.2.2 Territoriality

Following Zhou and Liu (2025), we used the 4-item scale of Avey et al. (2009) to assess employees’ territoriality. A sample item was “I feel I need to protect my ideas from being used by others in my organization” (α = 0.76).

#### 3.2.3 Self-interested voice

Following Liu et al. (2026), we used the 8-item scale of Duan et al. (2021) to measure self-interested voice. A sample item was “I communicate to the leader about my opinions only on issues relevant to my interests” (α = 0.89).

#### 3.2.4 Time urgency

Following Chen and Nadkarni (2017), we used the 6-item time urgency subscale from Landy et al. (1991) to measure time urgency. This measure has shown good reliability and validity and has been used in prior research (e.g., Mohammed and Nadkarni, 2011). A sample item was “I find myself hurrying to get places even when there is plenty of time” (*α* = 0.78).

#### 3.2.5 Perceived organizational politics

Following Guo et al. (2019), we assessed perceived organizational politics using the 6-item scale developed by Hochwarter et al. (2003). A sample item was “People do what’s best for them, not what’s best for the organization” (*α* = 0.93).

#### 3.2.6 Control variables

Because demographic characteristics have been shown to relate to employees’ attitudes and workplace behaviors (Bidwell and Briscoe, 2009), we controlled for participants’ age, gender, and education level in all subsequent analyses. Table 1 presents the means, standard deviations, and intercorrelations for all study variables.

**Table 1:** Descriptive statistics.

Variables	Mean	SD	VIF	1	2	3	4	5	6	7	8
1.Illegitimate Tasks	2.94	0.96	1.70	(0.94)							
2.Territoriality	3.78	0.70	1.05	0.13^*^	(0.76)						
3.Self-interested Voice	3.44	0.79	-	0.08	0.32^***^	(0.89)					
4.Time Urgency	3.36	0.70	1.05	0.19^**^	0.07	0.02	(0.78)				
5.Perceived Organizational Politics	2.72	0.98	1.68	0.62^***^	0.09	−0.07	0.16^**^	(0.93)			
6.Age	33.31	6.73	1.12	0.03	−0.16^**^	−0.05	0.07	0.03	-		
7.Gender	0.36	0.48	1.05	−0.03	−0.01	−0.08	−0.02	−0.12^*^	0.05	-	
8.Education level	3.82	0.58	1.15	0.05	0.06	0.08	−0.01	−0.05	−0.31^***^	0.15	-

*N* = 306; Cronbach’s alpha coefficients appear in parentheses along the diagonal; *p < 0.05, **p < 0.01, ***p < 0.001.

## 4 Data analysis and results

### 4.1 Common method bias

The degree to which common method bias may have influenced study findings was examined through Harman’s single-factor test (Podsakoff et al., 2003). The single largest factor emerging from this procedure explained 26.72% of the total variance, a figure that falls comfortably short of the 40% benchmark indicative of substantial bias. Additionally, inspection of the VIF values displayed in Table 1 confirmed that all VIF values were below 3. Taken together, these results did not indicate severe common method bias in this study.

### 4.2 Confirmatory factor analyses

Given the self-reported nature of all measures, a series of confirmatory factor analyses (CFAs) was conducted to assess construct distinctiveness. The CFA models were estimated using maximum likelihood estimation. Model fit was evaluated using χ^2^, df, CFI, TLI, RMSEA, and SRMR. As displayed in Table 2, the hypothesized five-factor model fit the data well (χ^2^/df = 2.12; CFI = 0.91; TLI = 0.90; RMSEA = 0.06; SRMR = 0.07) and performed better than competing alternative models, such as the single-factor model (χ^2^/df = 7.44; CFI = 0.47; TLI = 0.43; RMSEA = 0.15; SRMR = 0.16). Thus, these findings support the discriminant validity of the five constructs.

**Table 2:** Confirmatory factor analyses.

Factor Model	Factor Combination	χ^2^	df	χ^2^/df	CFI	TLI	RMSEA	SRMR
5-factor model	IT, TER, SIV, TU, POP	963.852	454	2.123	0.910	0.901	0.061	0.067
4-factor model	IT + TU, TER, SIV, POP	1331.320	458	2.907	0.845	0.832	0.079	0.083
4-factor model	IT + POP, TER, SIV, TU	1660.972	458	3.627	0.787	0.769	0.093	0.084
3-factor model	IT + TU + POP, TER, SIV	2021.431	461	4.385	0.723	0.702	0.105	0.096
2-factor model	IT + TU + POP, TER + SIV	2277.841	463	4.920	0.678	0.655	0.113	0.106
1-factor model	IT + TU + POP+TER + SIV	3451.435	464	7.438	0.470	0.433	0.145	0.162

+ represents factors merging into one. IT = Illegitimate tasks; TER = Territoriality; SIV = Self-interested voice; TU = Time urgency; POP = Perceived organizational politics.

### 4.3 Hypothesis testing

The hypotheses were tested using R 4.3.0 and the lavaan package with 5,000 bootstrap resamples (Rosseel, 2012). Before constructing the interaction terms, the composite scores of illegitimate tasks, time urgency, and perceived organizational politics were mean-centered. All reported coefficients are unstandardized. The results are presented in Table 3.

**Table 3:** Results of hypothesis tests.

Variables	Territoriality		Self-interested Voice
	Model 1	Model 2	Model 3
Intercept	3.99^***^ (0.41)	4.23^***^ (0.38)	1.58^**^ (0.51)
Age	−0.02^**^ (0.01)	−0.02^**^ (0.01)	0.00 (0.01)
Gender	0.00 (0.08)	−0.06 (0.08)	−0.15 (0.09)
Education level	0.01 (0.07)	0.01 (0.07)	0.11 (0.08)
Illegitimate tasks	0.10^*^ (0.04)	0.11^*^ (0.05)	0.03 (0.05)
Territoriality			0.35^***^ (0.06)
Time urgency		0.07 (0.06)	
Illegitimate tasks × Time urgency		0.24^***^ (0.05)	
Perceived organizational politics		−0.02 (0.05)	
Illegitimate tasks × Perceived organizational politics		0.06 (0.04)	
*R* ^2^	0.04	0.14	0.11

*N* = 306. *p < 0.05, **p < 0.01, ***p < 0.001.

H1 posited that illegitimate tasks would be positively related to employees’ territoriality, and Model 1 confirmed a significant path from illegitimate tasks to territoriality (*B* = 0.10, *SE* = 0.04, *p* < 0.05), supporting H1. For H2a, Model 3 showed that territoriality was positively associated with self-interested voice (*B* = 0.35, *SE* = 0.06, *p* < 0.001), and in line with H2b, territoriality mediated the effect of illegitimate tasks on self-interested voice (*indirect effect* = 0.04, 95% CI = [0.01, 0.08]). Thus, H2a and H2b were supported.

To test H3a and H4a, we simultaneously included both moderators and their respective interactions with illegitimate tasks in the same model. The interaction between illegitimate tasks and time urgency was positive and significant (*B* = 0.24, *SE* = 0.05, *p* < 0.001). To illustrate this moderation, we graphed the interaction (see Figure 2) and conducted simple slope analyses. Simple slope analyses showed that illegitimate tasks were positively associated with when time urgency was high (*simple slope* = 0.28, *t* = 4.48, *p* < 0.001), but not when time urgency was low (*simple slope* = −0.05, *t* = −0.77, *p* = 0.44). In contrast, the interaction between illegitimate tasks and perceived organizational politics was not significant (*B* = 0.06, *SE* = 0.04, *p* = 0.14). Thus, H3a was supported, whereas H4a was not supported.

**Figure 2:**
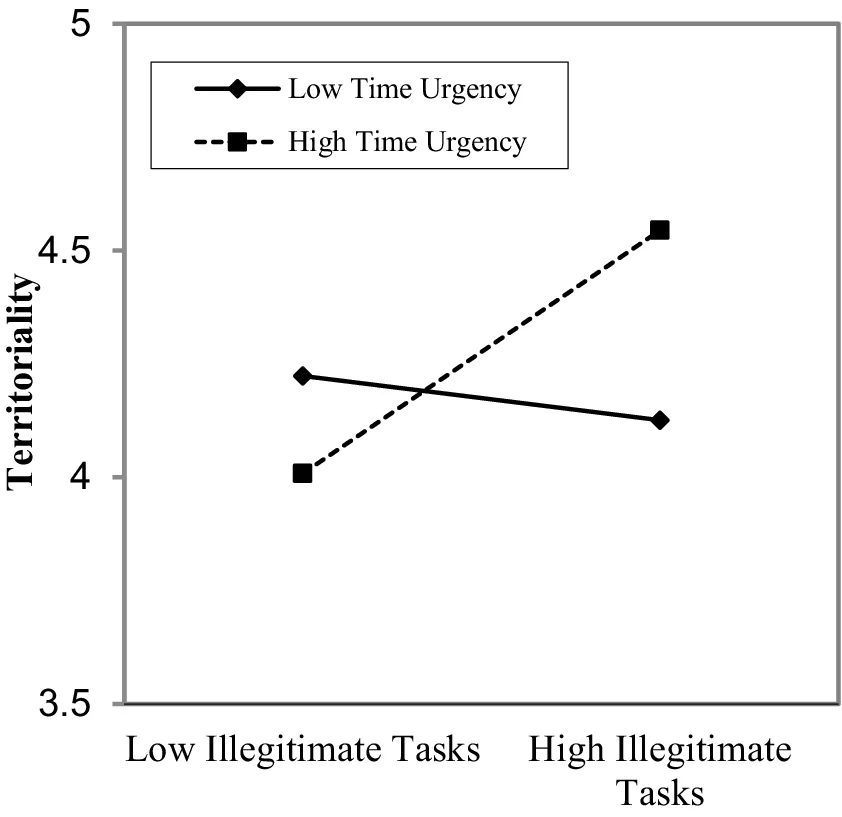
Interaction of IT and TU on territoriality. IT = Illegitimate tasks; TU = Time urgency.

To test H3b and H4b, we estimated a joint moderated mediation model in which time urgency, perceived organizational politics, and their respective interactions with illegitimate tasks were simultaneously included in the first-stage equation. The results are presented in Table 4. The indirect effect of illegitimate tasks on self-interested voice through territoriality was significant when time urgency was high (*indirect effect* = 0.10, *SE* = 0.03, 95% CI = [0.04, 0.17]), but nonsignificant when time urgency was low (*indirect effect* = −0.02, *SE* = 0.03, 95% CI = [−0.08, 0.04]). The index of moderated mediation for time urgency was significant (*Index* = 0.08, *SE* = 0.03, 95% CI = [0.03, 0.15]). In contrast, the indirect effect was significant when perceived organizational politics was high (*indirect effect* = 0.06, *SE* = 0.04, 95% CI = [0.004, 0.14]) but nonsignificant when perceived organizational politics was low (*indirect effect* = 0.02, *SE* = 0.03, 95% CI = [−0.03, 0.07]), the corresponding index of moderated mediation was not significant (*Index* = 0.02, *SE* = 0.02, 95% CI = [−0.009, 0.06]). Thus, H3b was supported, whereas H4b was not supported.

**Table 4:** Results of moderated mediation analysis.

Moderator	Conditional level/Test	Estimate	SE	95% CI
Time urgency	High (+1 SD)	0.10	0.03	[0.04, 0.17]
	Low (−1 SD)	−0.02	0.03	[−0.08, 0.04]
	Index of moderated mediation	0.08	0.03	[0.03, 0.15]
Perceived organizational politics	High (+1 SD)	0.06	0.04	[0.004, 0.14]
	Low (−1 SD)	0.02	0.03	[−0.03, 0.07]
	Index of moderated mediation	0.02	0.02	[−0.009, 0.06]

*N* = 306. Entries are unstandardized estimates. High and low moderator levels represent one standard deviation above and below the mean, respectively. Bootstrap standard errors and 95% confidence intervals were based on 5,000 resamples.

As supplementary analyses, we also examined the two moderators separately. The findings concerning time urgency remained significant. When perceived organizational politics was examined separately, its interaction with illegitimate tasks (*B* = 0.09, *SE* = 0.04, *p* = 0.03) and the corresponding index of moderated mediation (*Index* = 0.03, *SE* = 0.02, 95% CI = [0.003, 0.07]) were both significant. These results suggest that the predicted moderating pattern of perceived organizational politics emerged when it was examined separately but did not remain significant after the interaction involving time urgency was taken into account. However, because the separately estimated models did not account for the other moderator and its interaction term, we treated these results as supplementary and based our conclusions regarding the hypotheses on the joint model.

## 5 Discussion

Employing a three-wave time-lagged design, this study investigated a moderated mediation model in which territoriality served as a pathway connecting illegitimate tasks to self-interested voice. The results indicated that illegitimate tasks showed a significant positive link to employees’ territoriality, and those reporting elevated territoriality demonstrated a greater tendency toward self-interested voice. The joint analysis further showed that time urgency strengthened the first-stage path from illegitimate tasks to territoriality, making the indirect effect on self-interested voice more pronounced. Although perceived organizational politics showed a significant moderating effect when examined alone, this effect did not remain significant in the joint model; thus, H4a and H4b were not supported. These findings point to a more differentiated role of individual and context-referent boundary conditions in the resource-protective process examined in this study. In the next section, we discuss the theoretical and practical contributions of this study and propose directions for future research.

### 5.1 Theoretical contributions

First, this study advances research on illegitimate tasks by identifying territoriality as the underlying psychological mechanism linking illegitimate tasks to employee behavior, thereby introducing an explanatory perspective centered on prevention-focused psychological ownership. Prior research on illegitimate tasks has primarily examined employees’ reactions through the lens of incongruence between assigned tasks and professional roles, focusing on outcomes such as diminished work meaningfulness, reduced work motivation, heightened role conflict, and increased anxiety (Eatough et al., 2016; Semmer et al., 2015). Building on these existing frameworks, this study adds an explanatory perspective rooted in the resource-protective aspect of employees’ psychological ownership in the context of task assignment, revealing how illegitimate tasks are associated with territoriality by encroaching upon employees’ resource domains and subsequently drive their behavioral responses. This finding suggests that understanding the behavioral effects of illegitimate tasks requires attention not only to the legitimacy judgments triggered by task-role incongruence, but also to employees’ psychological responses at the level of resource ownership, which is an equally important pathway driving behavior.

Second, by identifying self-interested voice as an outcome variable through which illegitimate tasks influence employee behavior via territoriality, this study contributes to the literature on both illegitimate tasks and voice behavior. Recent studies have documented increasingly diverse behavioral responses to illegitimate tasks (Cheng et al., 2026; Ma et al., 2026; Tian et al., 2026; Zhao et al., 2022). Existing research on the interplay between illegitimate tasks and voice behavior has largely confined its attention to organization-oriented voice (Ouyang et al., 2025; Yuan et al., 2024; Zhao et al., 2022), with self-oriented voice receiving comparatively little scholarly attention. However, this study finds that the heightened territoriality associated with illegitimate tasks can drive employees toward self-interested voice as a means of actively securing favorable resource arrangements, rather than disengaging passively. In doing so, this study advances the theoretical understanding of the antecedents of self-interested voice by positioning it as a behaviorally motivated response to resource threat contexts (Duan et al., 2021; Morrison, 2011), while also broadening the examination of outcome variables associated with illegitimate tasks.

Finally, by jointly examining time urgency and perceived organizational politics, this study provides a more differentiated account of the boundary conditions surrounding the proposed resource-protective process. The two moderators represent theoretically distinct influences on resource-threat appraisal. Time urgency reflects an individual sensitivity to time scarcity (Burnam et al., 1975; Landy et al., 1991), whereas perceived organizational politics represents a broader, context-referent perception of how organizational decisions and resources are allocated (Ferris and Kacmar, 1992; Ferris et al., 1996). When both moderators and their interactions were estimated simultaneously, the findings supported time urgency, but not perceived organizational politics, as a boundary condition of the association between illegitimate tasks and territoriality; perceived organizational politics showed a significant moderating effect when examined in isolation. These contrasting patterns point to differentiated roles of individual and context-referent boundary conditions in the resource-protective process examined in this study.

One possible explanation lies in the relative proximity of the two moderators to the resource threat posed by illegitimate tasks. Illegitimate tasks impose immediate demands on employees’ time and attention, and time urgency captures individual sensitivity to precisely this form of resource loss (Burnam et al., 1975). Perceived organizational politics, by contrast, reflects a broader contextual perception of whether organizational decisions are driven by informal power and self-serving motives (Ferris and Kacmar, 1992; Ferris et al., 1996). When examined in isolation, perceived organizational politics may have captured employees’ general vigilance toward resource encroachment; once time urgency was simultaneously considered, however, this more threat-congruent individual characteristic may absorb part of the shared explanatory power, thereby limiting the incremental contribution of perceived organizational politics in the joint model. Consistent with COR theory’s emphasis on the value of resources and the threat of their loss (Halbesleben et al., 2014; Hobfoll, 1989), this interpretation suggests that the explanatory power of a boundary condition may depend partly on its congruence with the particular resource domain under threat. Accordingly, the contribution of the joint analysis lies not only in identifying time urgency as a boundary condition, but also in showing that theoretically plausible individual and context-referent boundary conditions may differ in their incremental explanatory value.

### 5.2 Practical implications

Beyond the theoretical contributions, the findings of this study also offer meaningful implications for organizational practice. First, at the level of task assignment, organizations should optimize task allocation and examine how illegitimate tasks are communicated and framed, with explicit attention to employees’ psychological perceptions of ownership over their own resources. Task allocation is not only an administrative decision; it is also a communication episode through which employees infer respect, fairness, and the allocation of valued resources. Managers should not treat illegitimate tasks as administratively neutral work directives but should instead recognize that such assignments carry an implicit threat to employees’ sense of ownership over resources such as their time, professional expertise, and energy. This sense of psychological encroachment may be associated with heightened territoriality, which, in turn, may be associated with subsequent defensive behavioral responses. The practical priority, therefore, is to proactively manage employees’ resource ownership experience throughout the task assignment process. Concretely, managers should ensure that tasks are necessary and reasonably distributed. When nonroutine or potentially illegitimate tasks cannot be avoided, managers should clarify their purpose, duration, and boundaries to prevent employees from interpreting them as a long-term appropriation of their resource domains. Organizations may help buffer employees’ territorial responses by offering compensatory resources such as additional support, flexible work arrangements, or future development opportunities. By focusing on the resource ownership dimension of task assignment, organizations can effectively reduce the psychological costs imposed by illegitimate tasks before defensive behavioral cycles take hold.

Second, at the level of behavioral monitoring, managers should broaden their interpretive lens when observing employees’ responses to illegitimate tasks and remain alert to the possibility that resource threat may manifest not as silence or withdrawal, but as self-interested voice. Shaped by prior research, managers’ intuitive expectations may lean toward anticipating disengagement or reduced voice among employees assigned illegitimate tasks (Ouyang et al., 2025). However, this study finds that when employees perceive external encroachment on valued work-related resources, they may instead turn to proactive self-interested voice, that is, raising suggestions primarily aimed at securing favorable resource arrangements rather than advancing the organization’s broader goals. If managers misread this self-interested voice as constructive engagement, they may inadvertently reinforce it; if they dismiss it as mere complaining, they may overlook the underlying signal of resource threat. Organizations should therefore train managers to distinguish self-interested voice from pro-organizational voice, and to treat the former as a diagnostic indicator of compromised resource ownership. When self-interested voice emerges, the appropriate managerial response is not suppression, but rather a genuine acknowledgment of the underlying territorial concerns regarding valued work-related resources, for instance by revisiting task boundaries, recognizing the burden the task imposes, or providing tangible resource support. In this way, organizations can redirect employees’ proactive coping energy toward more constructive ends.

Third, organizations should pay particular attention to employees’ time urgency when assigning illegitimate tasks. For employees with higher levels of time urgency, managers should prioritize a workload balance assessment before assigning illegitimate tasks, ensuring that the cumulative resource burden and the likelihood of defensive responses are minimized. Organizations can also establish structured workload review mechanisms that make the task allocation process transparent and negotiable, thereby reducing the perceived intensity of threat. Before making such assignments, managers can review employees’ existing workloads, clarify priorities and deadlines, explain why the task is necessary, and provide additional time or flexibility where possible. Transparent and consistent task-allocation procedures can further reduce ambiguity and help employees understand how and why responsibilities are assigned. Organizations should therefore combine fair and transparent allocation procedures with targeted support for employees whose time resources are most directly threatened by a task assignment. These practices are particularly important because employees high in time urgency are more sensitive to the time demands imposed by illegitimate tasks and may therefore be more likely to respond territorially. By addressing these time demands directly, organizations may reduce the pressure associated with illegitimate tasks and lower the likelihood of territoriality and subsequent self-interested voice.

### 5.3 Limitations and future research

First, although a three-wave design was adopted to support the proposed directional links, the observational nature of the study and reliance on employee self-reports limit our ability to draw causal conclusions. The temporal ordering does not rule out alternative explanations or reverse causal relationships among the variables. Future research should use multi-source data designs, experimental manipulations or cross-lagged longitudinal designs to address these limitations and strengthen causal inference. Second, we did not collect detailed information about participants’ industries or the size of their organizations. We therefore could not examine whether the proposed relationships varied across industries or between organizations of different sizes. Future research should collect these organizational characteristics and use stratified sampling to assess the generalizability of our findings across different industries and organizational contexts. Third, cross-cultural generalizability is constrained, as the sample was drawn solely from Chinese organizations characterized by high power distance and collectivist norms. Future research should replicate and extend this study in individualistic, low power distance settings to assess the cross-cultural generalizability of the proposed model. Finally, this study has certain limitations regarding the outcome variables examined and the boundary conditions explored. Regarding outcome variables, this study focused solely on self-interested voice; future research should examine broader self-protective responses, such as strategically withholding effort on illegitimate tasks. Regarding boundary conditions, future research could explore additional moderators at both individual and contextual levels, such as uncertainty avoidance (Jang et al., 2018) and team psychological safety (Edmondson, 1999), to delineate more precisely the boundary conditions of the proposed model.

## Data Availability

The raw data supporting the conclusions of this article will be made available by the authors, without undue reservation.
